# Dietary habits, physical activity, and self-reported rhinosinusitis in children and adolescents

**DOI:** 10.3389/fpubh.2023.1290307

**Published:** 2024-01-08

**Authors:** Katarzyna Pazdro-Zastawny, Joanna Krajewska, Mateusz Kolator, Alicja Basiak-Rasała, Sara Górna, Tomasz Zatoński

**Affiliations:** ^1^Department of Otolaryngology, Head and Neck Surgery, Wroclaw Medical University, Wroclaw, Poland; ^2^Department of Social Medicine, Wroclaw Medical University, Wrocław, Poland; ^3^Department of Physiology and Biochemistry, Poznan University of Physical Education, Poznań, Poland; ^4^Biegaj Dla Zdrowia Foundation, Wrocław, Poland

**Keywords:** physical activity, dietary patterns, sinusitis, adolescents, children

## Abstract

**Background:**

Pediatric paranasal rhinosinusitis is one of the more common pediatric diseases of the upper respiratory tract and it entails significant morbidity. Most commonly, it is caused by a viral infection of the nasal mucosa, which spreads through the natural passages within the cavities of the paranasal sinuses, leading to inflammation of the mucosa that lines the nasal cavity and paranasal sinuses.

**Methods:**

The objective of this cohort study was to assess whether there is a correlation between pediatric rhinosinusitis, physical activity, and selected dietary habits among pupils aged 6 to 16 years from elementary schools in Wrocław, Poland. This study – as part of the pro-health program “Let us Get the Kids Moving” – is also aimed at establishing factors that potentially predispose children to developing RS. The survey study was conducted on a group of 2,458 children and adolescents from elementary schools in Wrocław. The age of the examined children ranged from 6 to 17 years (mean = 10.8 years; standard deviation = 2.7).

**Results:**

Rhinosinusitis was more common in the children aged 13–17 years than in those aged 6–9 years (6.4% vs. 1.5%; *p* < 0.001) or 10–12 years (6.4 vs. 2.6%; *p* < 0.001). The study revealed a significant positive correlation between rhinosinusitis development and several variables: age > 11 years, attending swimming classes fewer than 1–2 times a week, using a computer, consuming milk, salty snacks, and carbonated sweet drinks, consuming fruit fewer than 1–2 times a week, not attending physical education classes, eating fewer than 4 meals, and not eating breakfast at home (*p* < 0.05).

**Conclusion:**

It is of great importance to establish preventive measures against recurrent upper respiratory tract infections that may predispose children to rhinosinusitis. Introducing healthier, traditional dietary habits and regular physical activity in children and adolescents may result in normal and adequate immune response and proper functioning of the inflammatory control system.

## 1 Introduction

Pediatric paranasal rhinosinusitis (RS) is one of the more common pediatric diseases of the upper respiratory tract and it entails significant morbidity. Most commonly, it is caused by a viral infection of the nasal mucosa which spreads through the natural ostia into the paranasal sinuses, leading to inflammation of the mucosa. The prevalence of acute RS in the pediatric population accounts for 6.5 to 13% of the patients with upper respiratory tract infections (URTI) (1). RS occurs as an effect of URTI in approximately 7.5% of children (2, 3). Its exact etiology remains unknown with viral, bacterial, or fungal infections, environmental irritants, allergies, and anatomical abnormalities being possible pathological factors. The characteristic symptoms of pediatric RS are nasal obstruction and/or excessive discharge of pathological secretions, pain or a feeling of pressure the face, and/or coughing, accompanied by characteristic endoscopic or sinus CT scan findings such as mucosal edema, nasal discharge, or nasal polyps. A diagnosis of acute RS, defined as an inflammation of the mucosal lining of one or more of the paranasal sinuses, is established when the aforementioned symptoms last less than 12 weeks, chronic RS can be diagnosed when the symptoms of sinus inflammation persist longer than 3 months despite proper standard treatment (4–6).

RS impairs self-perceived well-being in children and has a detrimental effect on children’s and adolescents’ quality of life because it may affect patients’ physical and mental health. It also has a negative impact on children’s learning and examination performance on exams. The consequences of rhinorrhea, congestion, and frequently associated symptoms such as sneezing, headache, fatigue, and lack of concentration may lead to sleep-disordered breathing and are connected to disturbances in learning performance, social relationships, mood, and attention. RS may result in sleep difficulties that lead to chronic daily fatigue, negatively influence studying process, and predispose to school absences. Thus, school performance in children and adolescents around the world is globally reduced (7–10).

The objective of the study was to assess if a correlation exists between pediatric chronic RS, physical activity (PA), and selected dietary habits in pupils ranged from 6 to 16 years from public schools in Wrocław, Poland and to establish the risk factors of developing chronic RS. The pro-health program called “Let us Get the Kids Moving” [“Uruchamiamy dzieciaki”] is a comprehensive survey study of children and adolescents with regard to their medical condition, the environmental and social factors that affect way of life, and the occurrence of variables associated with the development of selected non-communicable diseases.

## 2 Study design

The data showed in this study was acquired from the pro-health project “Let us Get the Kids Moving,” which was started in 2016 and was formed and arranged by the Wroclaw Medical University and the “Run for Health” foundation. “Let us Get the Kids Moving” Moving” is a school-based weight gain prevention program focus on parents, physical education teachers, and trainers through promotion of healthy lifestyle, as well as PA and a healthy food choice. It also aimed at drawing attention to the problem of excessive weight gain and evaluating factors and associated comorbidities, as well as educating teachers and parents. „Let us Get the Kids Moving” was designed to support school environments and families to focus on PA and a healthy diet. The program’s principal pillars comprise of: diagnostics, treatment, and prophylaxis. Wroclaw Medical University specialists created special questionnaire which was used in this survey study (the study questionnaire was included as an Appendix). School coordinators were responsible for distribution of questionnaires and handed out to children’s and adolescents’ caregivers during seasonal school interviews in the 2018–2019 and 2019–2020 school years. 31 elementary and junior high schools in Wroclaw obtained this parent-adolescent- reported questionnaire regarding chronic RS, PA, sedentary behaviors and dietary habits in children and adolescents. Regarding the fact that parents of older children and adolescents may not be completely aware of their dietary habits and/or what activities they are engaged in, in case of older children and adolescent’s questionnaire was completed together by a parent and their peer. The questionnaire was handed out to 10,000 families and the response rate was 33%.

A computer-generated program was used to randomly select 31 out of the 53 public schools in the city. All children from 7 to 18 years old who reside in Poland are entitled to free education in state-run school facilities. At the time of the study, the system of compulsory education included 1 year of preschool, 6 years of elementary school, 3 years of junior high school, and finally high school. These schools follow the Polish national curriculum and the government’s rules for assessing students established by the Ministry of Education. The part of the survey regarding PA consisted of questions concerning the child’s attendance in physical education classes at school. The participants were also asked to estimate the frequency of PA lasting at least 45 min in the last week; how much time per week the child spends doing popular sports, e.g., swimming; how much time per week is spent doing outdoor activities; how much time per week is spent on inactive behavior, e.g., sitting in front of a computer, TV, or smartphone; and whether the individual takes part in any additional organized sports activities outside of school. The survey also contained questions about comorbidities (including asthma, gastroesophageal reflux disease) dietary habits, namely, the number of meals, the consumption of snacks and breakfast, the eating of meals outside the home or school, and any specific diet that is followed. The diagnosis of chronic RS was defined as a physician diagnosed confirmed by otolaryngologist, multifactorial inflammatory and infectious process, covering the mucous membrane of the nose and paranasal sinuses. Confirmation of chronic RS symptoms included two or more of the following signs or symptoms for 12 weeks: nasal obstruction or nasal discharge of pathological secretions (anterior or posterior drip), pain or a feeling of pressure the face, and/or coughing. A positively worded questionnaire item with related numerical codes was used in this research.

The study was anonymous and voluntary. The Local Ethics Committee of Wrocław Medical University (KB-738/2018) approved the study. All children and adolescents and their caregiver took part in the project voluntarily and could discontinue their involvement at any time without consequences. The exact character and aim of the project was explained to all participants; in each case, consent to take part in the study was formally expressed. The ethical standards established in the 1964 Declaration of Helsinki and its later adjustments were a priority during conducting this study.

### 2.1 Data analysis

Statistical analyses were done using Dell Statistica 13.1 (TIBCO Software Inc., United States). The descriptive statistics are presented as mean values (M), standard deviation (SD), median values (Med), odds ratios (OR), and 95% confidence intervals (95% CI). The categorical variables were calculated as frequencies (percentages). The normality of distribution was determined using the Kolmogorov–Smirnov test. As there was a strong correlation between some of the independent (descriptive) variables – such as the child’s age, weight, and height – a multivariate logistic regression analysis was performed. For all tests, *p* < 0.05 was considered statistically significant.

## 3 Results

**Figure 1:**
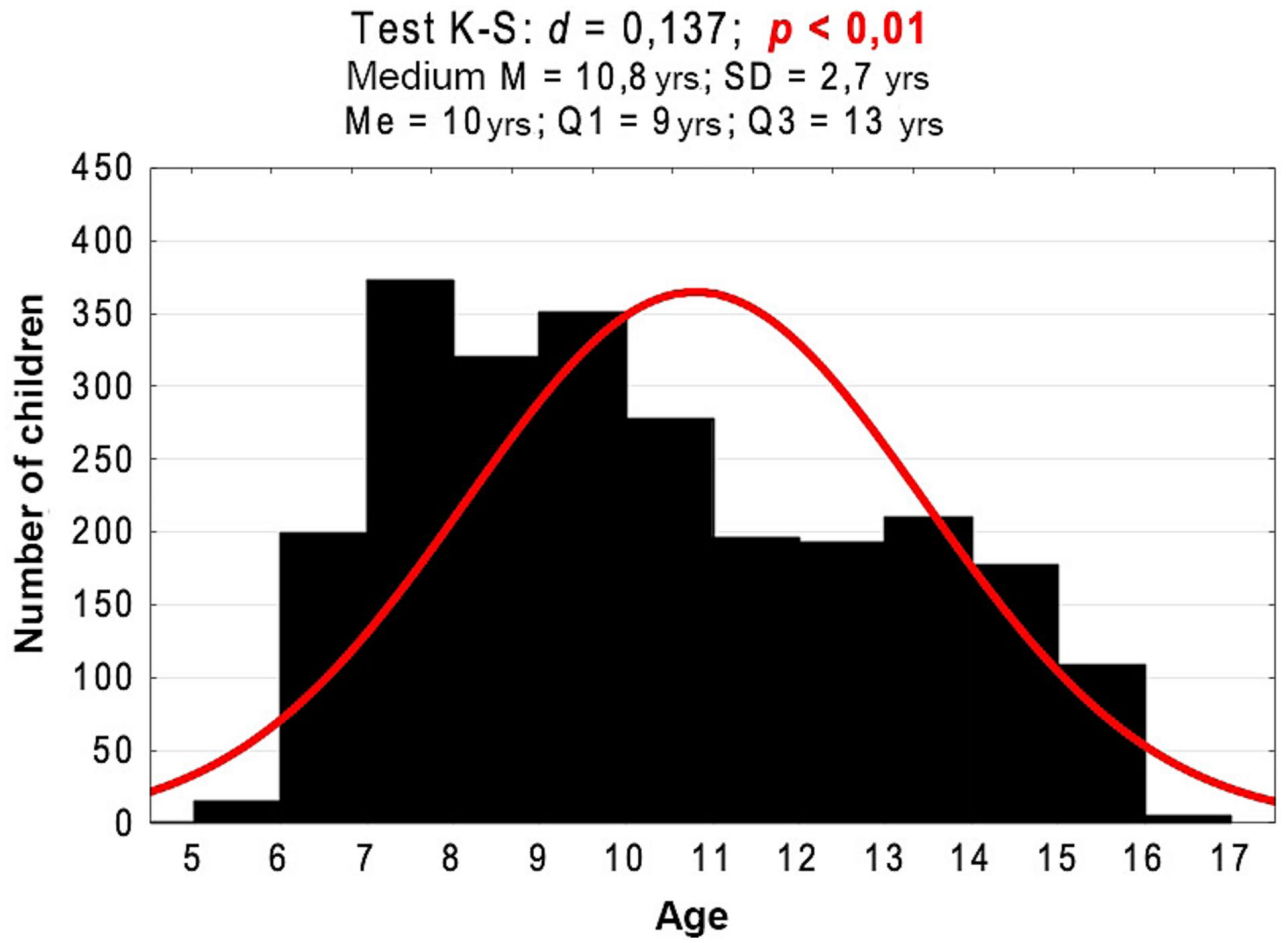
Age histogram of the examined children against a normal distribution and the results of the Kolmogorov–Smirnov normality test and basic descriptive statistics.

**Figure 2:**
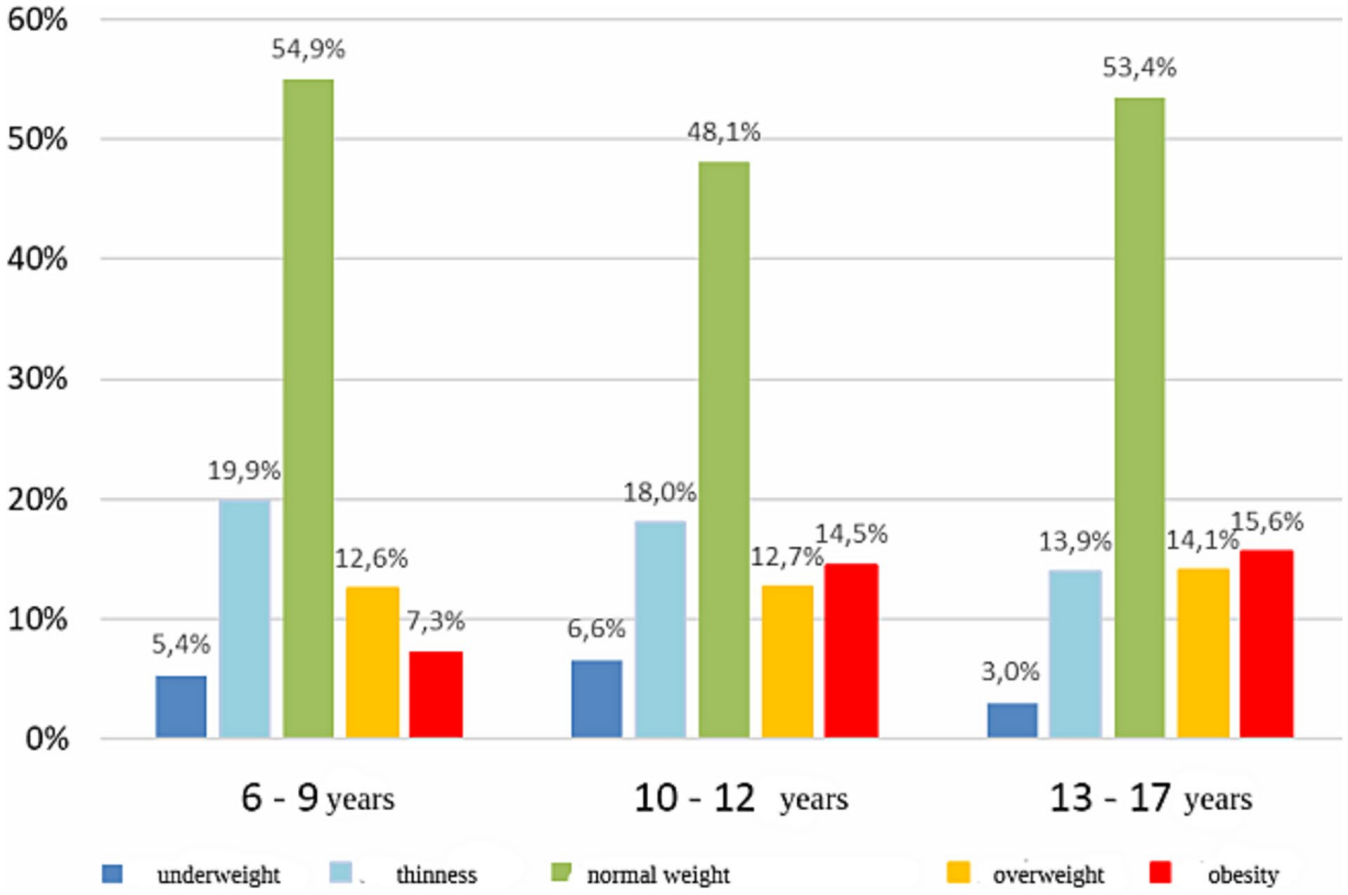
Percentage of examined children in subgroups differing in age and weight assessment.

The survey study was conducted among a group of 2,458 children and adolescents from primary schools in Wrocław. The research was conducted from 2018 to 2020 as part of the second round of the program “Let us Get the Kids Moving.” The age of the examined children ranged from 6 to 17 years (M = 10.8 years; SD = 2.7 years) (Figure 1). Among the examined children, girls constituted 50.6%. Pupils were divided into three following age groups. Group 1: children between 6 and 9 years of age, group 2: children aged 10–12 years, and group 3: children between 13 and 17 years of age. The division into three groups results from the structure of education in Poland. The youngest children covered by early childhood education: (children between 6 and 9 years of age), group two: children 10–12 years (teaching in grades 4–5) and group 3: children between 13 and 17 years of age-teaching divided into individual items. Based on the weight and height measurements of the children, the nutritional status index (Cole’s Index [CI]) was calculated. CI adjusts the BMI distribution for skewness and allows BMI in individual subjects to be expressed as an exact centile or SD score. The following interpretation of the CI was adopted: (1) 79% and less – underweight; (2) 80–89% – thinness; (3) 90–109% – normal weight; (4) 110–119% – overweight; and (5) 120% and above – obesity. The percentage of children with normal body weight in the age group of 10–12 years was significantly lower than in the age group of 6–9 years (48.1% vs. 54.8%; *p* = 0.005) and in the age group of 13–17 years (48.1% vs. 53.4%; *p* = 0.040) (Figure 2). The majority of surveyed children lived in 37.5% in the southern (37.5%) and eastern (28.5%) in eastern parts of the city. The incidence of chronic RS was not related to gender or place of residence (*p* > 0.05).

### 3.1 Rhinosinusitis

**Figure 3:**
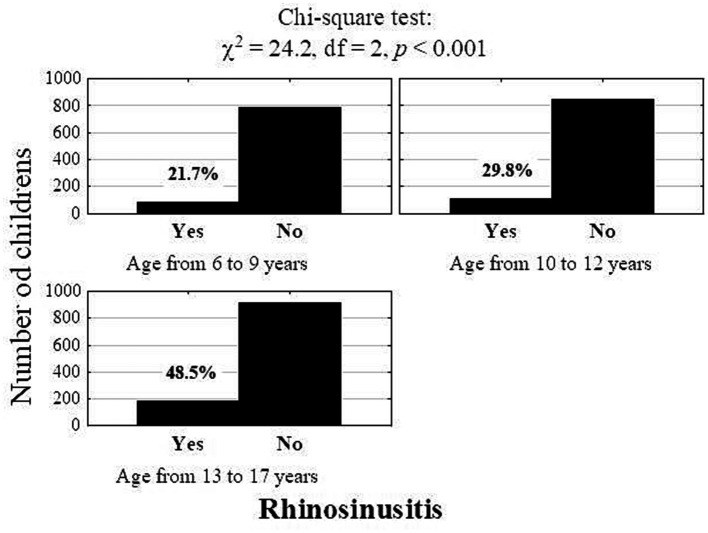
Number (percentage) of children with rhinosinusitis according to age (Pearson’s chi-square test).

**Figure 4:**
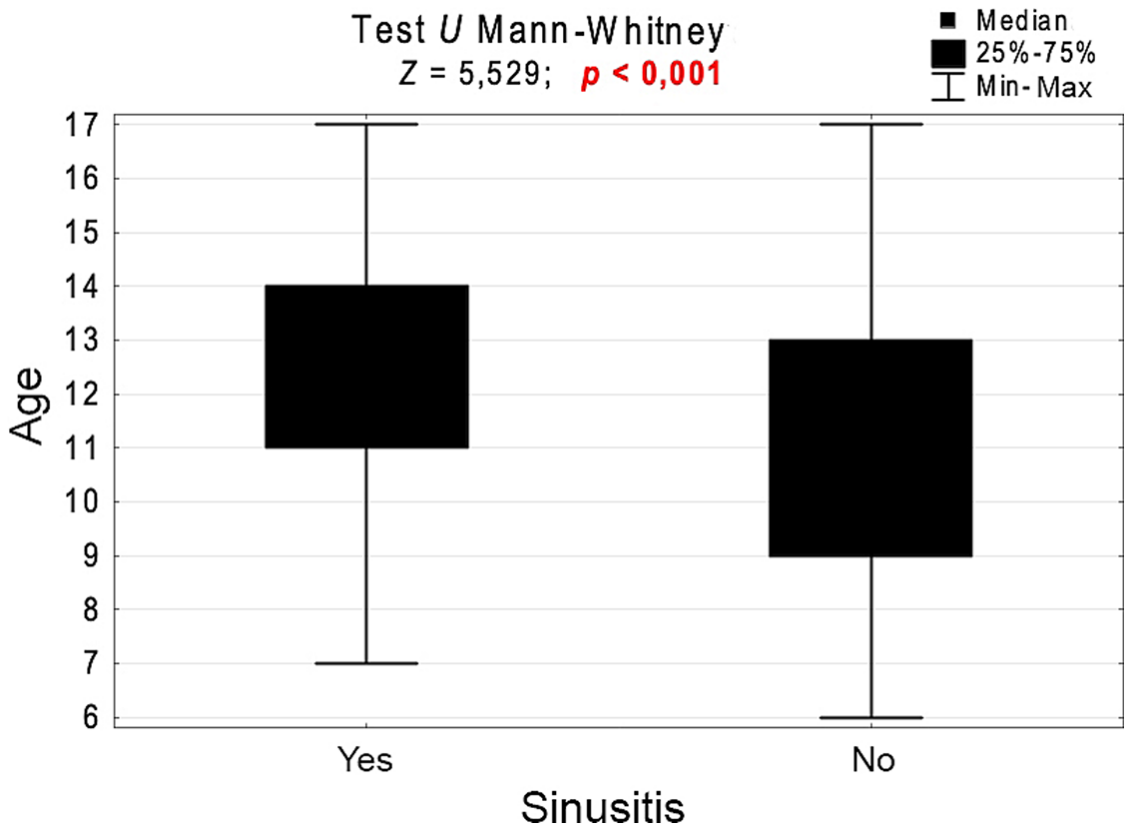
Age of children with and without rhinosinusitis and the results of the significance test.

**Figure 5:**
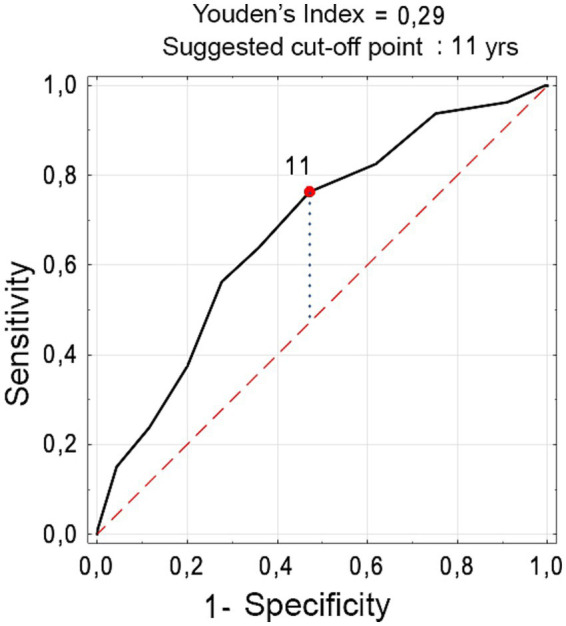
Receiver operating characteristic curve for estimating the chance of developing rhinosinusitis, based on the child’s Number (percentage) of children with rhinosinusitis according to age (Pearson’s chi-square test).

In the study group, the incidence of chronic RS significantly depended on the child’s age (*p* < 0.001, Figure 3). Chronic RS was more common in children aged 13–17 years than in those aged 6–9 years (6.4% vs. 1.5%; *p* < 0.001) or 10–12 years (6.4 vs. 2.6%; *p* < 0.001) (Table 1). Figures 4, 5 present the differences in the prevalence of chronic RS by age group, as well as the estimation of chronic RS prevalence by age (*p* < 0.01). A statistically significant correlation was found between age and the presence of chronic RS (*p* < 0.01). We found that children with chronic RS were older than those without RS. In our study, older children had a greater tendency to develop chronic RS (percentiles- the 25th, 50th [median], and 75th percentiles were lower for children without RS).

**Table 1:** Number (percentage) of children with rhinosinusitis, by age.

	6–9 years *N* = 919	10–12 years *N* = 835	13–17 years *N* = 704	*p*
Rhinosinusitis	14 (1.5%)	22 (2.6%)	45 (6.4%)	<0.001

The study revealed a significant positive correlation between RS development and several variables: age > 11 years, attending swimming classes fewer than 1–2 times a week, using a computer, consuming milk, salty snacks, and carbonated sweet drinks, consuming fruit fewer than 1–2 times a week, not attending physical education classes, eating fewer than 4 meals, and not eating breakfast at home (*p* < 0.05) (Tables 2, 3). The independent factors increasing the risk of RS were an age of 11 years or more, not taking part in physical education classes, eating fruit fewer than 5–6 times a week, and eating salty snacks at least 1–2 times a week (Table 4). The detailed analysis did not indicate statistically significant differences in the prevalence of asthma, allergic rhinitis, gastro-esophageal reflux disease and passive smoking between children with RS and those without (*p* values more than 0.05; data not presented). None of examined children and adolescents were active smokers.

**Table 2:** Variables significantly correlating with the presence of RS.

Variables correlating with RS	Rhinosinusitis (RS)		*p*
	Yes	No	
Age >11 years	12.5 ± 2.6	10.7 ± 2.6	<0.001
Attending swimming classes fewer than 1–2 times a week	1.05 ± 1.20	1.32 ± 1.07	0.003
Using a computer 3–4 hours or more on school days	1.07 ± 0.93	0.73 ± 0.73	0.001
Using a computer 3–4 hours or more on weekends and holidays	1.73 ± 0.91	1.38 ± 0.81	<0.001
Eating fruits fewer than 2 times a week	4.21 ± 1.61	4.53 ± 1.34	0.037
Consuming milk	3.84 ± 1.55	4.16 ± 1.45	0.049
Consuming salty snacks	2.16 ± 1.41	1.78 ± 1.23	0.007
Consuming carbonated sweet drinks	1.31 ± 1.36	1.01 ± 1.16	0.023

Quantitative variables are presented as means ± SD and qualitative variables as *n* (%). *p*, significance level; OR, odds ratio; 95% CI, 95% confidence interval.

**Table 3:** Comparison of various behavioral patterns in children with and without RS.

Variables correlating with RS	Rhinosinusitis (RS)		*p*	OR (95% CI)
	Yes	No		
Not attending physical education classes	76 (93.8%)	2303 (98.0%)	0.031	0.31 (0.12–0.80)
Eating <4–5 meals per day	24 (29.6%)	416 (18.0%)	0.008	1.92 (1.18–3.14)
Eating breakfast at home	56 (69.1%)	1884 (83.7%)	0.001	0.44 (0.27–0.71)

Quantitative variables are presented as means ± SD and qualitative variables as *n* (%). *p*, significance level; OR, odds ratio; 95% CI, 95% confidence interval.

**Table 4:** Logistic regression results for univariate and multivariate rhinosinusitis (RS).

Variables correlating with RS	Univariate model		Multivariate model		OR (95% CI)
	*b*	*p*	beta	*p*	
Age >11 years	1.281	<0.001	1.189	<0.001	3.28 (1.89–5.72)
Not attending physical education classes	1.171	0.016	1.226	0.016	3.41 (1.26–9.17)
Consuming fruit fewer than 5–6 times a week	0.551	0.020	0.558	0.027	1.75 (1.07–2.86)
Consuming salty snacks at least 1–2 times a week	0.701	0.004	0.648	0.014	1.91 (1.14–3.20)

Quantitative variables are presented as means ± SD and qualitative variables as *n* (%). *p*, significance level; OR, odds ratio; 95% CI, 95% confidence interval.

## 4 Discussion

Pediatric RS almost always occurs as a complication of common cold. Most commonly, a viral URTI causes RS secondary to edema of the nasal mucosa, overproduction and retention of secretions, impaired cilia function, obstruction of the sinus ostia, consequently leading to chronic inflammation of the mucosa covering the nasal cavity and the paranasal sinuses. The average child has between 6 and 8 episodes of colds annually, and it has been estimated that 5 to 10% of all URTI are complicated by RS (11, 12). Chronic RS in the pediatric population is multifactorial, and therefore requires a multifaceted, multidisciplinary approach. Understanding the complex etiology of this disease is crucial in choosing the optimal treatment. Chronic RS results from anatomical, histological, and immunological differences, as well as from the influence of specific factors predisposing them to the development of this disease.

It has been estimated that chronic RS is diagnosed in 2.1% of patients younger than 20 years in ambulatory health care visits per year (13) and the prevalence is increasing with age (14). RS develops in the most aired paranasal sinuses depending on their development and increasing size. The maxillary sinuses are the first of the paranasal sinuses to develop and grow progressively until the end of puberty. Small anterior ethmoid air cells are present at birth. There are two phases of rapid development of ethmoid sinus: during the first 2 years of life and before young adulthood. Complete development of ethmoid sinus occurs by the age of puberty. In school-aged children, the maxillary sinuses grow and reach full size around the age of 15, along with the sphenoid sinuses. The last to develop are the frontal sinuses – they are fully formed by the age of 21. The ostiomeatal complex is not fully developed in the youngest children. In our study, which included children ranged from 6 to 17 years of age, a statistically significant relationship between the occurrence of sinusitis and age was revealed. The age of 11 years was identified as a factor contributing to RS. Gilani et al. showed that children in the age group of 5 to 15-year-old are more likely to be affected by RS (13). Chronic RS is common disease in children at different ages (15) and affects both children and adolescents.

The biological pathway explaining the features of the chronic RS phenotype is characterized by continuous inflammation of the sinus mucosa. It can lead to its dysfunction and promote colonization of paranasal sinus with dysbiotic bacterial flora disturbing microbiome homeostasis of the sinuses. Due to the multifactorial inflammatory RS pathogenesis, with both individual and environmental factors, the importance of dietary modification as a complementary adjunct to existing medical therapies is emphasized. Specific dietary changes that contribute to inflammation reduction could be beneficial in chronic RS management and should be considered as a supplementary process in enhancing effects of conventional RS treatment (16).

In our study, the factors that significantly correlated with the occurrence of RS were eating fewer meals (<4), consuming salty snacks, consuming fruit less frequently, and consuming sugary carbonated drinks. RS may be promoted by an excessive body weight and/or the association of genetic, environmental, social and economic factors that may lead into its development (17).

In recent years, traditional diets enriched with fresh, plant-based foods have been gradually abandoned, while the consumption of animal-based and highly processed foods has increased. Individual dietary factors, the consumption of specific foods and dietary components, seem to have an effect on inflammation (18). The influence of dietary macronutrients like sugar and fats on inflammation induction through activation of Toll-like receptor 4 is emphasized. A diet low in fruits and vegetables affects the microbiome and changes both, the structure and function of the mucous membrane. Limiting proteins and fats in the diet by promoting a diet high in nondigestible carbohydrates may have a positive effect on the gut microbiome (16, 19–21).

The anti-inflammatory effects of a diet rich in fresh and seasonal foods; with a significant proportion of fruits, vegetables, legumes, and whole grains; olive oil, seeds, and nuts as the main sources of fat; limited consumption of proteins and fats of animal origin; and limited intake of precooked and industrial foods (e.g., the Mediterranean diet) have been observed in various studies (22, 23). Whole-grain foods which are rich in bioactive elements with anti-inflammatory properties, and as well as vegetables and fruits, are low-energy- dense foods with high concentrations of water, fiber, and plentiful in abundant antioxidant compounds and other anti-inflammatory phytonutrients (24–28). It has also been suggested that such a diet could control immune-based diseases (11, 19–22). The Mediterranean diet was recognized by the WHO as a model of a healthy diet for both children and adults. The basis of this diet is constituted by based on products with a low glycemic index, which come from a full milling such as whole grains. The incorporating into the diet a large number of natural products, has imparts antioxidant, chemo- preventive, and anti-inflammatory effects, and results in the reduction triglycerides and cholesterol levels, as well as postprandial glycaemia.

The incorporation of a traditional Mediterranean diet could be a major contribution to improvement in patients with recurring colds and frequent inflammatory complications. The limitation of products such as white bread, no home- made store bought pastries, cow’s milk, processed and red meats, carbonated drinks, and precooked fast food is connected with a notable reduction in episodes of recurrent URTI, its subsequent complications, and persistent nose obturation (29). An unsuitable dietary pattern may lead to changes in the mucosa that covers the nose and paranasal sinuses and may subsequently enhance its pro-inflammatory and hyper-reactive potential.

The quality of the diet may reduce the frequency of inflammatory processes in children. Thus, changing correcting certain nutritional mistakes in the patients’ diet may result in a significant reduction in URTI. An unsuitable dietary pattern may lead to changes in the mucosa that covers the nose and paranasal sinuses and may subsequently enhance its pro-inflammatory and hyper-reactive potential. Modification of nutritional habits may lead to reduction of recurrent RS episodes (30). González-Gil et al. evaluated the relationship between the consumption of food and the high-sensitivity C-reactive protein, which is the most commonly assessed inflammatory biomarker in clinical and epidemiological studies. It was revealed that a dietary pattern in European children with excessive consumption of sugar and processed products and rare limited consumption of vegetables and fruits in European children presenting excessive consumption of sugar and processed products, and limited consumption of vegetables and fruits is independently is independently related to increased levels of inflammatory markers. Thus, efforts should be made to improve the quality of the diet in children and adolescents in order to prevent future diseases related with chronic inflammation (31). Regular moderate PA can enhance the immune response (32). Physicians still forget to stress the importance of adequate levels of PA (33). A study performed prospectively in a large cohort of 1,509 Swedish men and women showed that high levels of PA (~1 h exercise/day) reduced the incidence of URTI (34). Our study showed positive correlation between RS development and sedentary behavior (namely using a computer, no adequate or lack of physical education). RS is a heterogenous and multifactorial disease, with both individual and environmental factors influencing its development. RS presents many similarities in etiology with asthma. It was revealed that regular PA is associated with reduced risk of exacerbations in women with asthma (35). Aerobic training has a positive effect on airway inflammation and remodelling, enhances respiratory mechanics and decreases Th2 immune response in a murine model of asthma. PA have a protective effect on the immune system’s activity and on the levels of several immune cells (36). PA have a protective effect on the immune system’s activity and on the levels of several immune cells (36). Moderate, habitual PA positively impacts health, potentially by inducing favorable changes in the immune system, such as increased levels of T cells, enhanced natural killer cells cytotoxicity and improved function of B cells, immunoglobulins and macrophages (37). It has been proposed that PA reduces not only eosinophil count in the airways, but also the expression of Th2 cytokines (IL-4, IL-5, and IL-13) (38–40). The beneficial effect of PA could also be related to a decreased expression of NF-κB and an increased expression of anti-inflammatory cytokines IL-10 and IL-1ra (38).

Obesity in children may be associated with the development of various otorhinolaryngologic diseases. Several studies revealed that high BMI was associated with the prevalence of chronic RS (32, 41). Obesity-induced metabolic changes and chronic systemic inflammation may compromise immunity. Increased production of cytokines, adipocytokines and adiponectin by the adipose tissue significantly leading to immune system dysfunction. Oxidative stress is considered as another factor linking obesity with inflammatory diseases. A study by Nam et al. (42) discussed the role of obesity in the development of chronic RS with nasal polyps. The study showed a distinct association between chronic RS with polyps and both, general and central obesity. The authors observed that the frequency of RS with polyposis in obese individuals was significantly higher than in those with normal weight. Additionally, the prevalence of RS-related olfactory dysfunction and purulent discharge were higher in the central obesity group than in controls. In contrast to that, Sidell et al. did not demonstrate an association between obesity and CRS (43). It was concluded that RS may be promoted by excessive weight, yet this was not confirmed by the results of the present study. The influence of obesity on sino-nasal inflammation requires further studies.

Our study did not demonstrate a relationship between pediatric chronic RS and its putative predisposing factors, such as asthma, allergic rhinitis (AR), gastro-esophageal reflux disease (GORD) and smoking. The presence of AR and asthma, may have an impact on clinical outcomes and contribute to the lack of disease control in chronic RS patients (44, 45). It has been also suggested that atopy predisposes to chronic RS’s development (46). Despite the fact that the incidence of chronic RS has been shown to occur more frequently in AR patients, a sound pathophysiologic mechanism has not been demonstrated, and consequently, the association between RS and AR remains a subject of debate (47, 48). Certain types of chronic RS including allergic fungal rhinosinusitis (AFRS) and central compartment atopic disease (CCAD) seem to be related with AR mostly. AR and chronic RS with nasal polyps may be linked as both of them are mediated by type 2 inflammatory response (49). RS and asthma share a close connection including common pathophysiological mechanisms. Mucosal cells infiltrations identified in RS and asthma share similar profile and present abundance of eosinophils, mast cells, macrophages, and T cells. Additionally, similar proinflammatory mediators including histamine, leukotrienes, interleukin 4, 5, and 13 are observed in sinonasal and bronchial mucosa (50). Chronic RS may complicate the diagnosis of asthma, and worsen both, its control and management (51). GORD has been indicated to be associated with chronic RS, but the strength of the connection is still discussed (52). The potential role of GORD in chronic RS development between may arise from the fact that GORD promotes direct exposure of the mucosa covering nasal cavity and nasopharyngeal to gastric acid, which leads to mucosal inflammation and impaired mucociliary clearance. It may consequently induce sinus ostia obstruction and predispose to recurrent infections. Also, malfunction of autonomic nervous system induced by gastric acid may cause spontaneous sinonasal mucosa edema and inflammation. The observed increased prevalence of *Helicobacter pylori* in the sinonasal mucosa in patients with chronic RS may also highlight the potential role of GORD in chronic RS development (53). In our study we did not find correlation between GORD and pediatric chronic RS. Several reviews also did not reveal such association (54–56). In our study none of children and adolescents were active smokers. Studies suggest that there is an association between tobacco smoke exposure and chronic RS highlighting influence of active smoking usage and the development of this condition and suggesting a dose dependant effect (the number of cigarettes) and the prevalence of chronic RS. Tobacco smoking may predispose to chronic RS development via inducing sinonasal irritation and congestion, rhinorrhea, and increased nasal airway resistance. It also alters sinonasal epithelial mucociliary clearance and negatively influences innate immune function (57).

In our study children and adolescents in our study represented schools from all districts of the city of Wrocław and included children from all parts of the city. Despite the fact that some parts of Wrocław were more widely represented, the individual districts the city are evenly distributed, taking into account socioeconomic conditions.

## 5 Strengths and limitations

We did not find other research that would evaluate the correlation between various dietary and physical behaviors and chronic RS in children; thus, we were unable to juxtapose our results with others. The limitation of this study is the lack of accompanying medical information used in the diagnosis of RS, such as endoscopic findings and computerized tomography – which provides detailed images of the sinuses – or information regarding the use of medication lowers the quality of evidence. However, access to medical records concerning RS was not a part of the study. The children’s and adolescent’s complaints and were self-reported by their parents or legal guardians. RS symptoms were also self-reported and consequently there may be issues about the accuracy of the diagnosis. The other limitation is the lack of information about the use of medication, related or not to chronic RS.

## 6 Conclusion

It is of great importance to establish preventive measures against recurrent URTI that may predispose a person to RS. Introducing healthier, traditional dietary habits and regular physical activity in children and adolescents may result in normal and adequate immune response and proper functioning of the inflammatory control system that guards the human body.

## Data availability statement

The raw data supporting the conclusions of this article will be made available by the authors, without undue reservation.

## Ethics statement

The studies involving humans were approved by The Local Ethics Committee of Wrocław Medical University/KB-738/2018/. The studies were conducted in accordance with the local legislation and institutional requirements. Written informed consent for participation in this study was provided by the participants’ legal guardians/next of kin.

## Author contributions

KP-Z: Data curation, Formal analysis, Investigation, Resources, Validation, Visualization, Writing – original draft. JK: Data curation, Project administration, Resources, Writing – review & editing. MK: Methodology, Resources, Software, Writing – review & editing. AB-R: Resources, Validation, Writing – review & editing. SG: Data curation, Investigation, Writing – review & editing. TZ: Conceptualization, Data curation, Formal analysis, Resources, Writing – review & editing.
